# Super High Contrast USPIO-Enhanced Cerebrovascular Angiography Using Ultrashort Time-to-Echo MRI

**DOI:** 10.1155/2024/9763364

**Published:** 2024-04-13

**Authors:** Liam Timms, Tianyi Zhou, Ju Qiao, Codi Gharagouzloo, Vishala Mishra, Rita Maria Lahoud, John W. Chen, Mukesh Harisinghani, Srinivas Sridhar

**Affiliations:** ^1^Department of Physics, Northeastern University, Boston, MA, USA; ^2^Department of Mechanical and Industrial Engineering, Northeastern University, Boston, MA, USA; ^3^Gordon Center for Medical Imaging, Massachusetts General Hospital, Boston, MA, USA; ^4^Department of Radiology, Massachusetts General Hospital, Boston, MA, USA; ^5^Department of Chemical Engineering, Northeastern University, Boston, MA, USA; ^6^Department of Bioengineering, Northeastern University, Boston, MA, USA; ^7^Theranano LLC, Newton, MA, USA

## Abstract

**Background:**

Ferumoxytol (Ferahame, AMAG Pharmaceuticals, Waltham, MA) is increasingly used off-label as an MR contrast agent due to its relaxivity and safety profiles. However, its potent T2^∗^ relaxivity limits achievable T1-weighted positive contrast and leads to artifacts in standard MRI protocols. Optimization of protocols for ferumoxytol deployment is necessary to realize its potential.

**Methods:**

We present first-in-human clinical results of the Quantitative Ultrashort Time-to-Echo Contrast Enhanced (QUTE-CE) MRA technique using the superparamagnetic iron oxide nanoparticle agent ferumoxytol for vascular imaging of the head/brain in 15 subjects at 3.0T. The QUTE-CE MRA method was implemented on a 3T scanner using a stack-of-spirals 3D Ultrashort Time-to-Echo sequence. Time-of-flight MRA and standard TE T1-weighted (T1w) images were also collected. For comparison, gadolinium-enhanced blood pool phase images were obtained retrospectively from clinical practice. Signal-to-noise ratio (SNR), contrast-to-noise ratio (CNR), and intraluminal signal heterogeneity (ISH) were assessed and compared across approaches with Welch's two-sided *t*-test.

**Results:**

Fifteen volunteers (54 ± 17 years old, 9 women) participated. QUTE-CE MRA provided high-contrast snapshots of the arterial and venous networks with lower intraluminal heterogeneity. QUTE-CE demonstrated significantly higher SNR (1707 ± 226), blood-tissue CNR (1447 ± 189), and lower ISH (0.091 ± 0.031) compared to ferumoxytol T1-weighted (551 ± 171; 319 ± 144; 0.186 ± 0.066, respectively) and time-of-flight (343 ± 104; 269 ± 82; 0.190 ± 0.016, respectively), with *p* < 0.001 in each comparison. The high CNR increased the depth of vessel visualization. Vessel lumina were captured with lower heterogeneity.

**Conclusion:**

Quantitative Ultrashort Time-to-Echo Contrast-Enhanced MR angiography provides approximately 5-fold superior contrast with fewer artifacts compared to other contrast-enhanced vascular imaging techniques using ferumoxytol or gadolinium, and to noncontrast time-of-flight MR angiography, for clinical vascular imaging. This trial is registered with NCT03266848.

## 1. Background

Cerebrovascular disease is the most common life-threatening neurological event in the developed world. Most cerebrovascular diseases are accompanied by structural abnormalities of the head and brain vessels, so improving safe imaging techniques to examine these abnormalities is crucial for clinical practice. Magnetic resonance angiography (MRA) has become a vital clinical tool to visualize and assess vascular abnormalities and cerebrovascular pathologies without invasive procedures or iodinated contrast.

Time-of-flight (TOF) MRA provides inflow-dependent enhancement of blood moving into the imaging volume without the injection of an exogenous contrast agent (CA) and is the most common approach for routine angiographic evaluation [1]. However, distortions or signal loss due to susceptibility effects and abnormal vessels with a turbulent flow can produce misleading TOF images, implying abnormalities where none exists or hiding those which are most severe [2]. In addition, the dependence of TOF contrast on blood flow direction, velocity, and relative depth into the imaging slab limits coverage of the cerebrovasculature and can necessitate multiple scans to capture vessels of interest across the whole head and brain.

Contrast-enhanced magnetic resonance angiography (CEMRA) using an exogenous CA is a well-established approach for noninvasive imaging. The most widely deployed CAs are gadolinium (Gd) based (GBCAs). Recently, GBCAs were used with caution in patients with impaired kidney function due to the risk of nephrogenic systemic fibrosis [3, 4]. Yet, while nephrologist guidelines continue to recommend Gd avoidance in kidney disease patients [5, 6], the use of macrocyclic agents has largely alleviated this risk. However, additional concerns have been raised regarding the long-term accumulation of Gd throughout the body and brain [7], which occurs even in patients with healthy renal function [8]. GBCA-delivered Gd accumulation has not been associated with adverse health outcomes in humans, but this remains an active research area. There remains an impetus to produce a CEMRA technique without Gd in this context.

The potential of ultrasmall superparamagnetic iron-oxide nanoparticle, ferumoxytol, as an alternative CA in patients with kidney dysfunction has been previously recognized [9–11]. As a result, it has been frequently used off-label for MRI [12]. However, most studies utilize black-blood T2-weighted techniques, limiting their clinical applications, or bright-blood T1-weighted (T1w) sequences, whose achievable positive contrast is limited by susceptibility artifacts [13] and T2^∗^ weighting.

We translated the Quantitative Ultrashort Time-to-Echo (UTE) Contrast-Enhanced (QUTE-CE) MRA technique for cerebrovascular imaging from preclinical models to humans and provided comparisons to related techniques, including TOF MRA and blood pool contrast-enhanced (both ferumoxytol and Gd) T1w images. We hypothesized that the QUTE-CE MRA protocol would yield higher positive contrast with ferumoxytol, enabling a new approach to clinical CEMRA. This enabled the assessment of the QUTE-CE MRA technique using ferumoxytol for vascular mapping across the head and brain in a first-in-human clinical trial.

## 2. Methods

### 2.1. Study Cohort

This investigation was a prospective study registered under NCT03266848 on ClinicalTrials.gov. All procedures were institutional review board-approved and compliant with the Health Insurance Portability and Accountability Act. Volunteers were screened for MR safety. Participants provided written informed consent for the imaging procedure. Ferumoxytol (Ferahame, AMAG Pharmaceuticals, Waltham, MA) was infused, under observation, external to the MR scanner with a total 510 mg dose of elemental iron in 11 participants and 3 mg/kg of body weight in 4 participants. A subset of 5 participants also underwent abdominal scans [14].

The inclusion criteria for potential subjects were (1) age of 18-80 years old and (2) able to understand written consent documents and HIPAA authorization prior to initiation of study-related procedures by passing a Mini-Mental State Exam. Exclusion criteria were (1) known allergy to ferumoxytol or any intravenous iron preparation, (2) iron saturation above the upper limit of normal, (3) a contraindication to MRI, (4) known clinical conditions with a risk of iron overload, and (5) any medical condition which the investigators believed would have increased risk for an adverse event.

### 2.2. Imaging Protocol

Data were acquired at the Massachusetts General Hospital (MGH) Athinoula A. Martinos Center for Biomedical imaging on a 3T scanner (MAGNETOM Prisma, Siemens Healthcare, Erlangen, Germany). Each participant was imaged in two separate sessions: (1) precontrast, prior to the infusion, and (2) postcontrast, within 3.5 hours of the infusion. In all but one participant, both sessions were conducted within 8 hours. Data were acquired with a Siemens 64-channel head coil for thirteen participants, while a 32-channel head coil was used for two participants.

QUTE-CE MRA scans were optimized following the procedure in Gharagouzloo [15] and performed using a Siemens Works In Progress: UTE Spiral VIBE sequence. This sequence provides a nonslice-selective 3D excitation, a minimum TE of 0.05 ms, and a stack-of-spirals trajectory during readout. The flip angle was adjusted as necessary for SAR limitations, while TE, TR, and resolution remained fixed. The parameters for UTE scans were TR = 3.1 ms, minTE = 0.05 ms, FA = 15-18°, and FOV = 256 × 256 × 256 mm with a scan time of 6:36 min. In the precontrast session, TOF was acquired with a 65 mm thick imaging slab centered on the circle of Willis with a superior saturation band. TOF images were acquired with TR = 21 ms, TE = 3.44 ms, and FA = 20° with a scan time of 6:20 minutes. Standard TE T1-weighted (T1w) Magnetization Prepared RApid Gradient Echo (MPRAGE) was acquired in both sessions with TR = 2.31 s, TE = 2.9 ms, TI = 0.9 s, and FA = 9° and scan time of 5:29 minutes with the same FOV as the UTE scans. All images were reconstructed/resliced to 1 mm isotropic resolution. In 1 participant, blood was drawn preceding and proceeding with CA infusion. These blood vials were positioned near a bullet-shaped Siemens 5300 ml MR phantom of nickel sulfate hexahydrate and imaged with the same protocol. All scans were examined for severe motion artifacts and excluded or repeated when necessary.

Additional anonymized Gd MPRAGE images using Dotarem (Guerbet, Paris, France) were obtained retrospectively from current clinical practice but were not acquired in this study. These images were captured with TR = 2.33 s, TE = 2.32 ms, *TI* = 0.9 s, and FA = 8° with a scan time of 9:46 min and FOV = 256 × 256 × 256 mm. They were acquired in the blood pool phase approximately 8 minutes after the initial administration of the CA.

### 2.3. Image Preprocessing

Images were preprocessed with a pipeline implemented with Nipype [16] using functions from SPM [17], FSL [18], ANTs [19], and custom code. Images were bias-field corrected with the N4ITK algorithm [20], and Gibbs-ringing was minimized with subvoxel shifts [21]. After motion correction and registration, the brain was segmented using FSL's skull-stripping function with manual refinement in the slicer [22]. Finally, images were examined by JC and MH (combined 30+ years of clinical experience).

### 2.4. Image Segmentation and Metric Quantification

Regions of interest (ROIs) were segmented semiautomatically for quantification. Frangi et al.'s vesselness maps [23] were computed for TOF and QUTE-CE MRA using the Vascular Modeling Toolkit [24]. Vesselness maps were computed with values from 0.9 to 1.0 in large vessel lumina. Intraluminal vessel ROIs were segmented with a threshold of 0.95 vesselness. ROIs to sample the air and tissue were drawn manually with a slicer. The standard deviation of the signal in the air was used to estimate thermal noise. Ex vivo, ROIs were drawn manually in the precontrast blood sample, postcontrast blood sample, and the air.

These ROIs were used to calculate (1) blood signal-to-noise ratio (SNR), (2) contrast-to-noise ratio (CNR) between blood and tissue (blood-tissue CNR), (3) CNR between precontrast and postcontrast images (pre-post CNR), and (3) intraluminal signal heterogeneity (ISH). SNR was defined as the mean intensity in an ROI divided by the thermal noise measured from the air standard deviation. Blood-tissue CNR was defined as the difference between the blood SNR from tissue SNR in the postcontrast scan. The pre-post CNR was defined as the SNR difference between a postcontrast scan and SNR in the same ROI as a coregistered precontrast scan. Finally, the ISH was defined as the ratio of the standard deviation to the mean signal intensity in the same luminal ROI [25].

### 2.5. Statistical Tests

Statistics were calculated with the Pandas (1.2.1) and Scipy (1.6.0) Python libraries. Seaborn (0.11.1) was used for all plotting. Welch's two-sided *t*-test was used to calculate all comparison *p* values with *p* < 0.05 designated as the rejection of the null hypothesis. All values are provided with mean ± standard deviation where possible.

## 3. Results

### 3.1. Participant Characteristics

Fifteen participants (54 ± 17 years old, 9 women) were imaged from November 2017 to July 2020. No volunteers experienced adverse reactions to the procedure. UTE and MPRAGE images were acquired pre- and postcontrast infusion in 15 participants, while TOF images were acquired precontrast in 12 participants.

### 3.2. QUTE-CE MRA Provides the Highest Vessel Contrast

**Figure 1:**
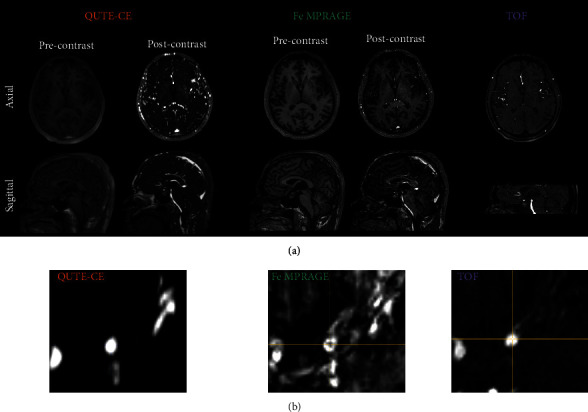
QUTE-CE provides a high vascular signal and contrast with low heterogeneity. (a) Typical raw axial and sagittal slices of Quantitative Ultrashort Time-to-Echo Contrast-Enhanced MR angiography (QUTE-CE MRA), ferumoxytol contrast-enhanced Magnetization Prepared RApid Gradient Echo (Fe MPRAGE), and time-of-flight (TOF) MRA. All slices are taken from the same participant after alignment and coregistration, allowing visual comparison of the same anatomical structures in each modality. QUTE-CE MRA captures more vessels than the other techniques. (b) Cropped views of an exemplary luminal cross-section from each technique.

**Figure 2:**
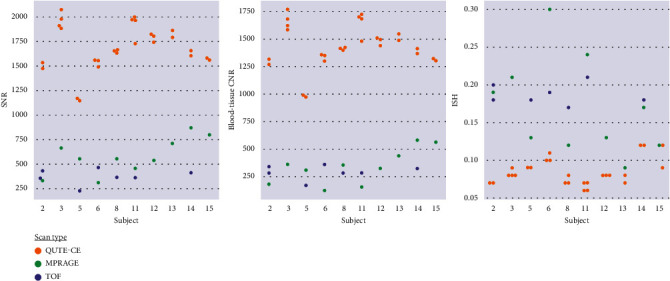
Signal-to-noise ratio (SNR), intraluminal signal heterogeneity (ISH), and contrast-to-noise ratio (CNR) for participants with directly comparable scans. Quantitative Ultrashort Time-to-Echo Contrast-Enhanced MR angiography (QUTE-CE MRA) provides higher blood SNR and blood-tissue CNR than the time-of-flight (TOF) MRA or T1-weighted ferumoxytol contrast-enhanced Magnetization Prepared RApid Gradient Echo (Fe MPRAGE) in every participant while maintaining lower ISH. Participants are excluded in cases where scans were significantly motion-degraded.

The postcontrast QUTE-CE yielded high-quality CEMRAs of the human head (Table 1 and Figure 1). Per-participant comparisons for each metric are presented in Figure 2. On average, across all participants, QUTE-CE MRA yielded an SNR of 4.98 times higher (3.3-5.3 per participant) than the TOF MRA (*p* < 0.001). The measured blood-tissue CNR was 5.34 (3.7-5.8 per participant) times higher in QUTE-CE than TOF (*p* < 0.001). QUTE-CE MRA provided an average of 3.6 (1.9-4.9 per participant) times higher luminal SNR, 5.8 (2.33-10.7 per participant) times the blood-tissue CNR, and 3.2 (1.7-6.9 per participant) the pre-post CNR relative to the standard TE T1w MPRAGE sequence. These differences all had *p* < 0.001. The SNR values for Fe MPRAGE and TOF were statistically different (*p* = 0.008), while blood-tissue CNR values were not (*p* = 0.40). Ex vivo results are provided in Supplemental Digital Content Figure S1.

### 3.3. QUTE-CE MRA Results in a More Homogeneous Lumen

Figure 1 provides an example of the same vessel lumen in coregistered views of the 3 different techniques in one participant. The lumen is most clearly and brightly delineated using QUTE-CE MRA with the least heterogeneity. In comparison, the other two techniques have signal variations arising from susceptibility artifacts for Fe MPRAGE and flow dependency for TOF MRA. This qualitative difference is captured quantitatively with ISH. The average ISH was 2.04 times lower in the QUTE-CE images than the TOF (Table 1). Relative to the standard T1w Fe MPRAGE, QUTE-CE MRA provided a 2.5-fold reduction in ISH (Table 1). Figure 2 provides per-participant comparisons of measured ISH values. QUTE-CE ISH was significantly different from both TOF (*p* < 0.001) and Fe MPRAGE (*p* < 0.005), while Fe MPRAGE and TOF ISH values were not (*p* = 0.44).

### 3.4. QUTE-CE MRA Yields High-Quality Visualization of the Entire Vasculature

**Figure 3:**
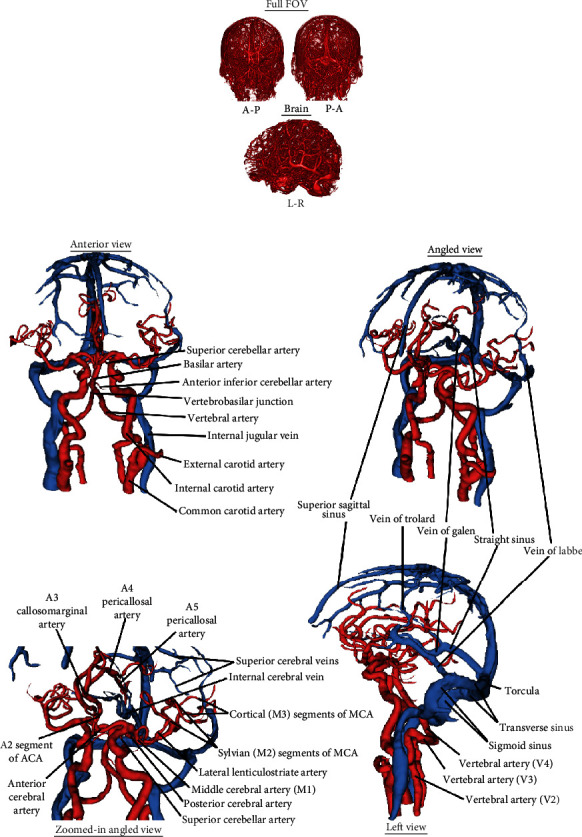
QUTE-CE provides detailed vascular imaging in a single scan. (a) Detailed 3D surface renderings of Quantitative Ultrashort Time-to-Echo Contrast-Enhanced MR angiography (QUTE-CE MRA) postcontrast image intensity for a participant; full head in front-facing anterior-posterior (A-P) direction, head in back-facing posterior-anterior (P-A) direction, and brain in left-facing left-right (L-R) view. (b) Select vascular structures segmented from QUTE-CE images with annotation of major arteries (red) and veins (blue).

Figure 3 displays representative 3D cerebrovascular renderings. Postcontrast images were acquired in the blood pool phase, 1-3 hours after infusion. QUTE-CE MRA yields bright-blood 3D images of the entire vasculature within the field of view, including both arteries and veins. A full 3D rendering is presented in Supplemental Digital Content Video S1. The clarity and definition of the raw-intensity images are apparent in these renderings. We did not measure significant differences between the image quality metrics of the two dosing regimens (510 mg vs. 3 mg/kg) used in the study; SNR (*p* = 0.74), ISH (*p* = 0.11), blood-tissue CNR (*p* = 0.74), or post-pre CNR (*p* = 0.89). The high contrast was robust to this variation in the experimental design (Supplemental Digital Content Figure S2).

### 3.5. QUTE-CE MRA Produces Fundamentally Different Images than TOF and GBCA Imaging

**Figure 4:**
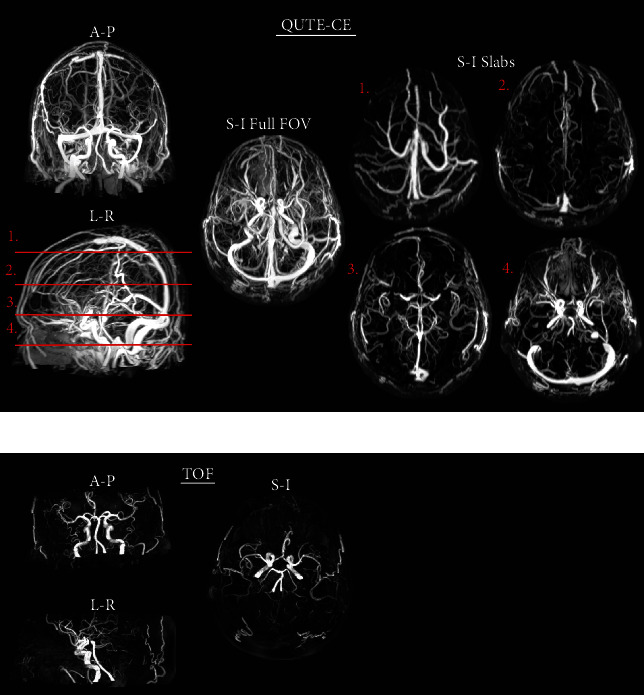
The technique provides more detailed vascular images than time-of-flight (TOF). Maximum intensity projection (MIP) (a) Quantitative Ultrashort Time-to-Echo Contrast-Enhanced (QUTE-CE) and (b) TOF MR angiography image intensities demonstrating the complete contrast enhancement of the vasculature in QUTE-CE MR angiography. QUTE-CE MR angiography captures all vasculature available in the TOF and much more of the cerebral vascular system in approximately the same imaging time. This detail can be examined by subsectioning the QUTE-CE field-of-view into slabs, labeled in the left-right MIP of the full field-of-view and presented as S-I projections of each slab.

In Figure 4, representative 3D maximum intensity projections (MIPs) are presented for QUTE-CE MRA and TOF MRA from the same participant. QUTE-CE MRA includes all vasculature present in the TOF and provides more of the cerebral vascular system in approximately the same imaging time. However, multiple arterial and venous TOF MRA scans would be needed to achieve the full field-of-view visualized here. Nevertheless, TOF MRA does not typically achieve the depth of vessel visualization of QUTE-CE MRA due to lower CNR and signal loss as a function of depth into the imaging slab at 3T.

**Figure 5:**
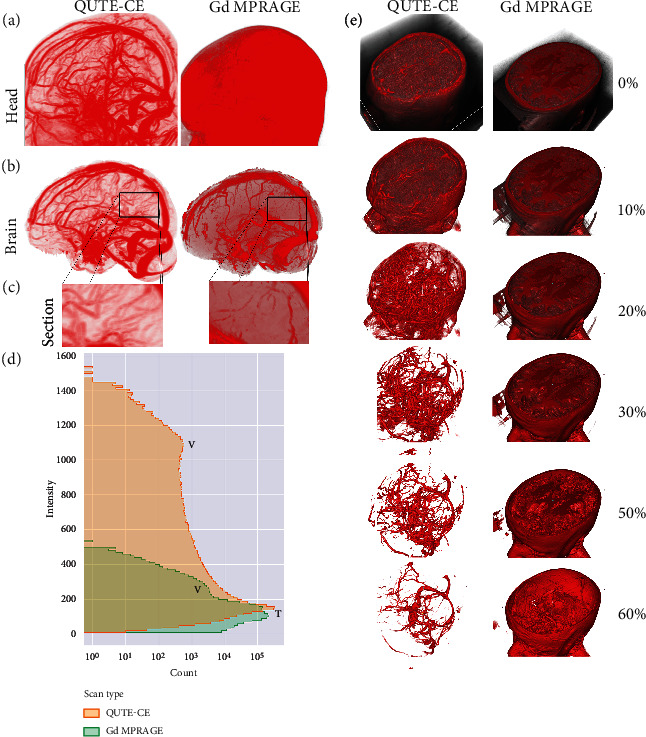
The technique provides qualitatively and quantitatively different vascular images than blood pool GBCA imaging. (a) Quantitative Ultrashort Time-to-Echo Contrast-Enhanced MR angiography compared to gadolinium-based contrast-agent enhanced Magnetization Prepared RApid Gradient Echo (MPRAGE), presented in maximum intensity projections (MIPs) from different individuals. High SNR and broad suppression of background (tissue, fat, and skull) signals enable simple visualization of the intracranial vascular network. (b) To examine the intracranial vessels, postprocessing is required to strip the skull while preserving superior cortical vessels for Gd MPRAGE. (c) Focusing on a cutout section emphasizes the increased delineation of smaller vessels using QUTE-CE MRA. (d) Histograms of raw intensity values in the cropped brains for the two techniques. Histograms capture the order of magnitude difference between the vessels (V) and tissue (T) in the two techniques. (e) As the high QUTE-CE CNR results in vascular enhancement below the voxel size, changing the visible intensity threshold allows examining vasculature across sizes. This effect is not possible with standard blood pool Gd imaging. Note that the top of the skull is cropped from both images to enable visualization of the structures inside the head in the Gd MPRAGE scan.

In Figure 5, QUTE-CE MRA is compared to Gd MPRAGE, presented in MIPs of two participants. QUTE-CE's background signal suppression throughout the head enables direct visualization of the intracranial vascular network without requiring complex processing and skull-stripping. Conversely, in Gd MPRAGE, the equivalent vessels are obscured due to background signals closer to the same values. In the Gd MRPRAGE, the measured blood SNR was 291, with a blood-tissue CNR of 168 and ISH of 0.15.

To examine the intracranial vessels in Gd MPRAGE equivalently to QUTE-CE MRA, the skull must be stripped while preserving superior cortical vessels, as shown in Figure 5(b). Nevertheless, even with equivalent skull stripping, QUTE-CE MRA enables the visualization of additional smaller vessels. There is a broader dynamic range of vascular enhanced intensities in the QUTE-CE image, resulting in an order of magnitude extended range of intensity values dominated by vascular signal (histograms Figure 5). Due to the distribution of vessel intensities over this wide dynamic range, varying the threshold of visible intensity values directly varies the range of visible vessel sizes in QUTE-CE MRA. This concept is presented in Figure 5(e). Supplemental Digital Content Video S2 provides a dynamic visualization of the same concept utilizing varying thresholds.

## 4. Discussion

Quantitative Ultrashort Time-to-Echo Contrast-Enhanced MR angiography is a method for vascular imaging consisting of a UTE pulse sequence optimization combined with a strong T1-shortening intravascular contrast agent. We translated QUTE-CE MRA from preclinical development [15, 26] to a clinical trial in humans and produced highly detailed vascular images of all vessels in the human head compared to conventional techniques. The technique yielded exceptionally high positive contrast bright blood across the field of view while minimizing or eliminating common artifacts.

The method captures extensive vascular detail without requiring a long acquisition time, nephrotoxic contrast agents, ultra-high-field machines, or complex processing. The images contain a wide dynamic range of intensity values dominated by vascular signals (Supplemental Digital Content Figure S3). Vessels below the voxel size provide sufficient contrast for visualization. This high CNR effect explains the richer detail captured compared to other approaches at identical resolution. The angiograms here were obtained in single 6:36-minute scans of the participants. Obtaining comparable visualizations using other MRA techniques requires multiple scans, increasing exam, and postprocessing time.

QUTE-CE MRA provides superior image quality to TOF MRA. While expected due to the use of the CA, this comparison is helpful as TOF is the current standard of care for MRA without GBCAs. Thus, QUTE-CE MRA has utility over TOF when greater detail, full 3D cerebrovascular coverage, flow-independent vascular information, or CEMRA without GBCAs is required. However, the administration of any contrast agent is a notable drawback. The technique is therefore complementary to routine noncontrast methods.

The technique provides higher contrast than standard TE T1w imaging with ferumoxytol. The potential for ferumoxytol CEMRA using traditional T1w blood pool contrast-enhanced imaging was previously identified in several studies, including children [27] and CKD patients [28]. However, earlier work primarily utilized traditional MRI sequences and relied on the dilution of ferumoxytol to avoid T2^∗^ signal suppression. The reliance on these standard sequences with a typical TE of about 3 ms led to prior conclusions that ferumoxytol could not deliver the same CNR as GBCAs [25], capturing signal at UTE overcomes this restriction. The exceptionally high CNR achieved in this study can be attributed to three main reasons: (1) the strong T1-shortening effect of the blood caused by the blood pool contrast agent; (2) the strong T1 weighting from the optimal choice of high flip angle and short TR; (3) the less T2^∗^ decay from the short TE with the UTE sequence. Though only a few feasibility studies combining UTE with ferumoxytol have been completed in humans, the results of our study agree closely with the results from Knobloch et al. [29]. They utilized the combination for pulmonary CEMRA and reported superior image quality relative to both Gd CEMRA and standard TE ferumoxytol CEMRA. The high-contrast angiograms also enable the delineation of all cranial vessels and quantitative examinations of the change in lumen size (Supplemental Digital Content Figure S4).

The potential advantages of this technique over the gold-standard GBCA in the blood pool phase are due to two principal factors. First, the intravascular nature of ferumoxytol eliminates tissue enhancement observed with extracellular GBCAs. Second, the protocol optimization, which constitutes the QUTE-CE technique, theoretically maximizes luminal intensity while minimizing luminal heterogeneity. Traditional blood pool phase GBCA imaging results in a lower dynamic range between blood and tissue signal intensities, obscuring vessel visualization and tracking with background signals. While this obfuscation can be reduced with additional postprocessing, the lower blood-tissue CNR still delivers less vascular detail.

QUTE-CE MRA offers an alternative approach to venography with advantages over other techniques. For example, CT venography requires the use of iodinated contrast agents, which are also contraindicated for participants with compromised kidney function. Further, it requires the removal of bone from the images by graded subtraction that is not needed here. In noncontrast-enhanced approaches, TOF requires additional scans for venography, whereas QUTE-CE MRA renders veins fully in the blood pool phase. Thus, QUTE-CE MRA venography has potential applications in various venous conditions like dural venous sinus thrombosis or stenosis, possible occlusion due to tumor invasion, vascular malformations, and idiopathic intracranial hypertension.

This approach is also without risk of nephrotoxicity or brain deposition. Recent studies have shown no deposition in the brain from ferumoxytol infusion [30, 31]. In addition, while an FDA black-box warning was issued for ferumoxytol in 2015, there have been no severe adverse reactions since [12], likely due to changes in the standard infusion procedure.

### 4.1. Limitations

There were a few limitations to our study. First, some images were excluded due to participant motion. Motion artifacts were not confined to a single modality and affected multiple approaches. The motion was mitigated in later participants with additional head padding. Second, this study did not capture first-pass images during the arterial phase of ferumoxytol as our participants were infused externally to the MRI scanner due to logistic constraints. Separation of arteries and veins is possible with first-pass imaging during contrast infusion and is now used with QUTE-CE MRA in an ongoing study [14]. Artery-vein segmentation may also be achieved in postprocessing, as shown successfully in CT angiography [32].

## 5. Conclusions

The image quality measured here, and the alternative safety profile of the CA may support the potential clinical deployment of Quantitative Ultrashort Time-to-Echo Contrast-Enhanced MR angiography for blood pool contrast-enhanced MR angiography. The QUTE-CE MRA technique was assessed to provide high-quality luminal enhancement and measurements of vascular morphometry, relative to conventional techniques. This may potentially be of great clinical use in diagnosing and monitoring cerebrovascular diseases such as stroke, carotid stenosis, vertebral stenosis, intracranial stenosis, thrombosis embolism hemorrhage, aneurysms, and vascular malformations. In addition, the technique may provide the basis for novel biomarkers for the characterization of etiologies or the detection of patterns of vascular diseases. These advantages of Quantitative Ultrashort Time-to-Echo Contrast-Enhanced MR angiography are not restricted to the head and brain. Indeed, we hypothesize they may be successfully utilized across the entire vasculature of the human body.

## Figures and Tables

**Table 1:** Image quality metrics measured from Quantitative Ultrashort Time-to-Echo Contrast-Enhanced MR angiography (QUTE-CE MRA), ferumoxytol contrast-enhanced Magnetization Prepared RApid Gradient Echo (Fe MPRAGE), time-of-flight (TOF) MRA, and gadolinium (Gd) contrast-enhanced MPRAGE.

Scan type	*N* scans	SNR	ISH	Blood-tissue CNR	Post-pre CNR
Current study					
QUTE-CE	27	1707 ± 226	0.091 ± 0.031	1447 ± 189	1460 ± 242
Fe MPRAGE	13	551 ± 171	0.186 ± 0.066	319 ± 144	454 ± 184
TOF	10	343 ± 104	0.190 ± 0.016	269 ± 82	—
Clin. practice					
Gd MPRAGE	1	291	0.150	168	—

“*N* scans” is the total number of images used for metric quantification across participants.

## Data Availability

All data required to assess the results and conclusions are provided in this paper, including Supplementary Materials. Deidentified anonymized images are available from the corresponding author by request. All image processing libraries used are open source. Requests related to the data processing pipeline details and implementation are available from Liam Timms.

## Abbreviations

CA: Contrast agent
TOF: Time-of-flight
GBCA: Gadolinium-based contrast agent
UTE: Ultrashort time-to-echo
QUTE-CE: Quantitative Ultrashort Time-to-Echo Contrast-Enhanced
CNR: Contrast-to-noise ratio
ROI: Region of interest
SNR: Signal-to-noise ratio
ISH: Intraluminal signal heterogeneity
CEMRA: Contrast-enhanced MRA.

## Supplementary Materials

Supplementary Materials Figure S1: ex vivo results. Figure S2: results are robust to the variations in study design. Figure S3: intensity histograms for QUTE-CE, Fe MPRAGE, and TOF per subject. Figure S4: diameters of vessel segments of interest can be quantitatively assessed. Video S1: 3D rendering. Video S2: dynamic thresholding.
