# Supplementary material for: Super High Contrast USPIO-Enhanced Cerebrovascular Angiography Using Ultrashort Time-to-Echo MRI
Source: Int J Biomed Imaging. 2024 Apr 13;2024:9763364. doi: 10.1155/2024/9763364 (PMC11032209; doi:10.1155/2024/9763364)
Supplement: Supplementary Materials — Figure S1: ex vivo results. Figure S2: results are robust to the variations in study design. Figure S3: intensity histograms for QUTE-CE, Fe MPRAGE, and TOF per subject. Figure S4: diameters of vessel segments of interest can be quantitatively assessed. Video S1: 3D rendering. Video S2: dynamic thresholding. [file 9763364.f1.docx]

Supplementary Materials for

**Super High Contrast USPIO-Enhanced Cerebrovascular Angiography Using Ultrashort-Time-to-Echo MRI**

Liam Timms, Tianyi Zhou, Ju Qiao, Codi Gharagouzloo, Vishala Mishra, Rita Maria Lahoud, John Chen, Mukesh Harisinghani, and Srinivas Sridhar*

*Corresponding author. Email: s.sridhar@northeastern.edu

**This file includes:**

Figs. S1 to S4

Captions for Videos S1 to S2

**Other Supplementary Materials for this manuscript include the following:**

Videos S1 to S2

**Supplementary Figure 1**


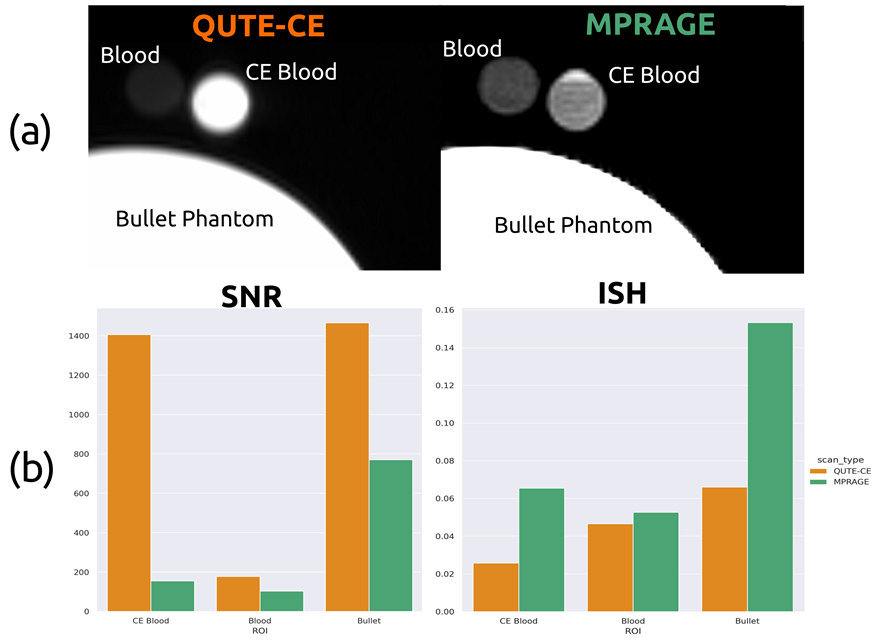


**Fig. S1. Ex vivo results.** (a) From left to right-axial cross-sections of the tubes consisting of blood taken prior to the infusion, contrast-enhanced blood (CE blood) taken just after the infusion, and a standard Siemens bullet shaped phantom; (b) SNR values for ROIs drawn in each tube, QUTE-CE provides higher contrast-enhancement; ISH values calculated within the tubes (rather than in vivo lumina) showing lower heterogeneity of the QUTE-CE signal relative to a standard T1w scan.

**Supplementary Figure 2**


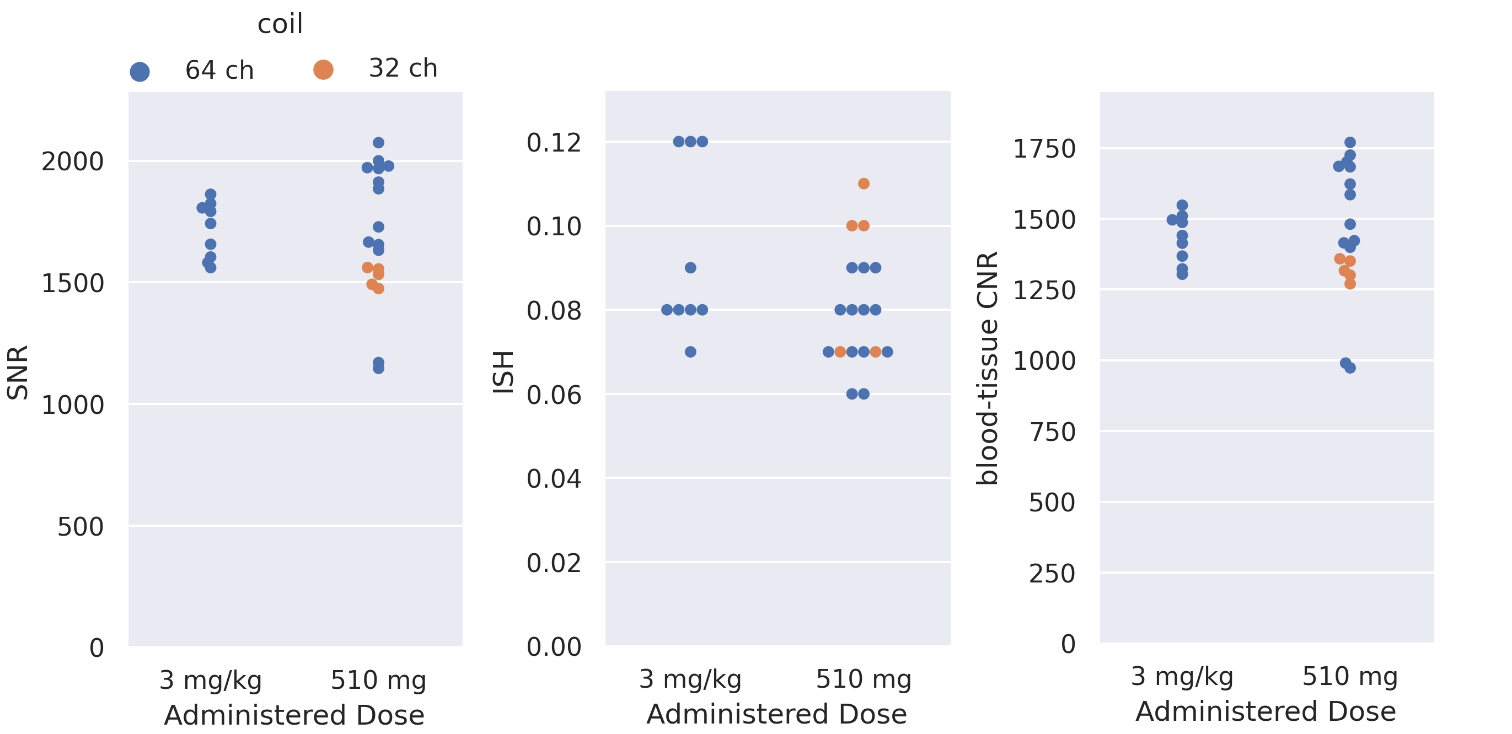


**Fig. S2. Results are robust to the variations in study design.** From left-to-right SNR, ISH and blood-tissue CNR for the two dosing regimes and the coils. The high SNR and CNR and low ISH were robust to these variations in study design with no statistically significant difference between dosing regimes (p-values in Results).

**Supplementary Figure 3**


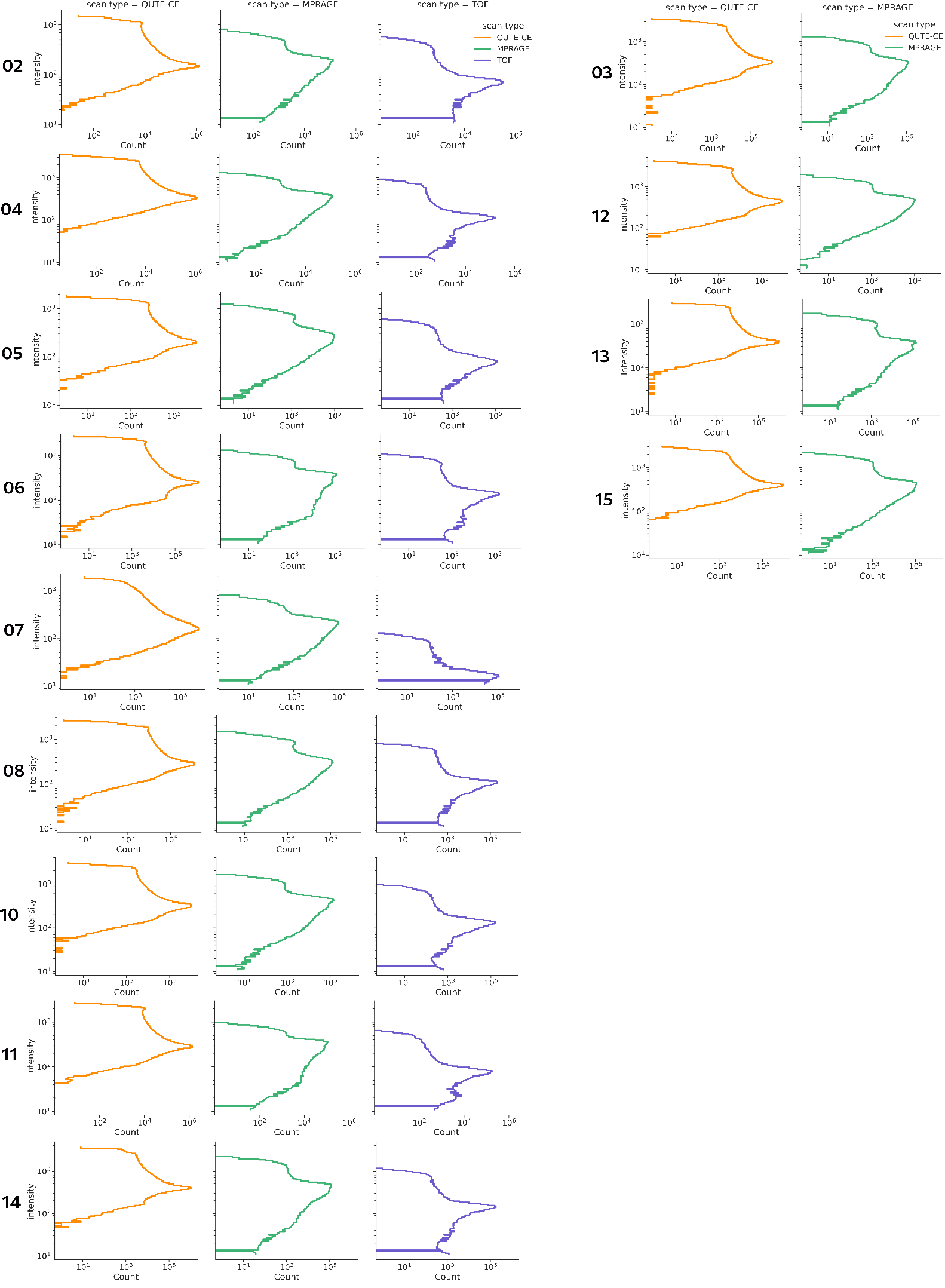


**Fig. S3. Intensity Histograms for QUTE-CE, Fe MPRAGE, and TOF per-subject.** From left-to-right; QUTE-CE (orange), Fe MPRAGE (green) and TOF (blue) intensity values within the cropped brain. From top-to-bottom; each scan modality’s corresponding histogram for 12 subjects. TOF data is omitted for subjects where it was not collected.

**Supplementary Figure 4**

**
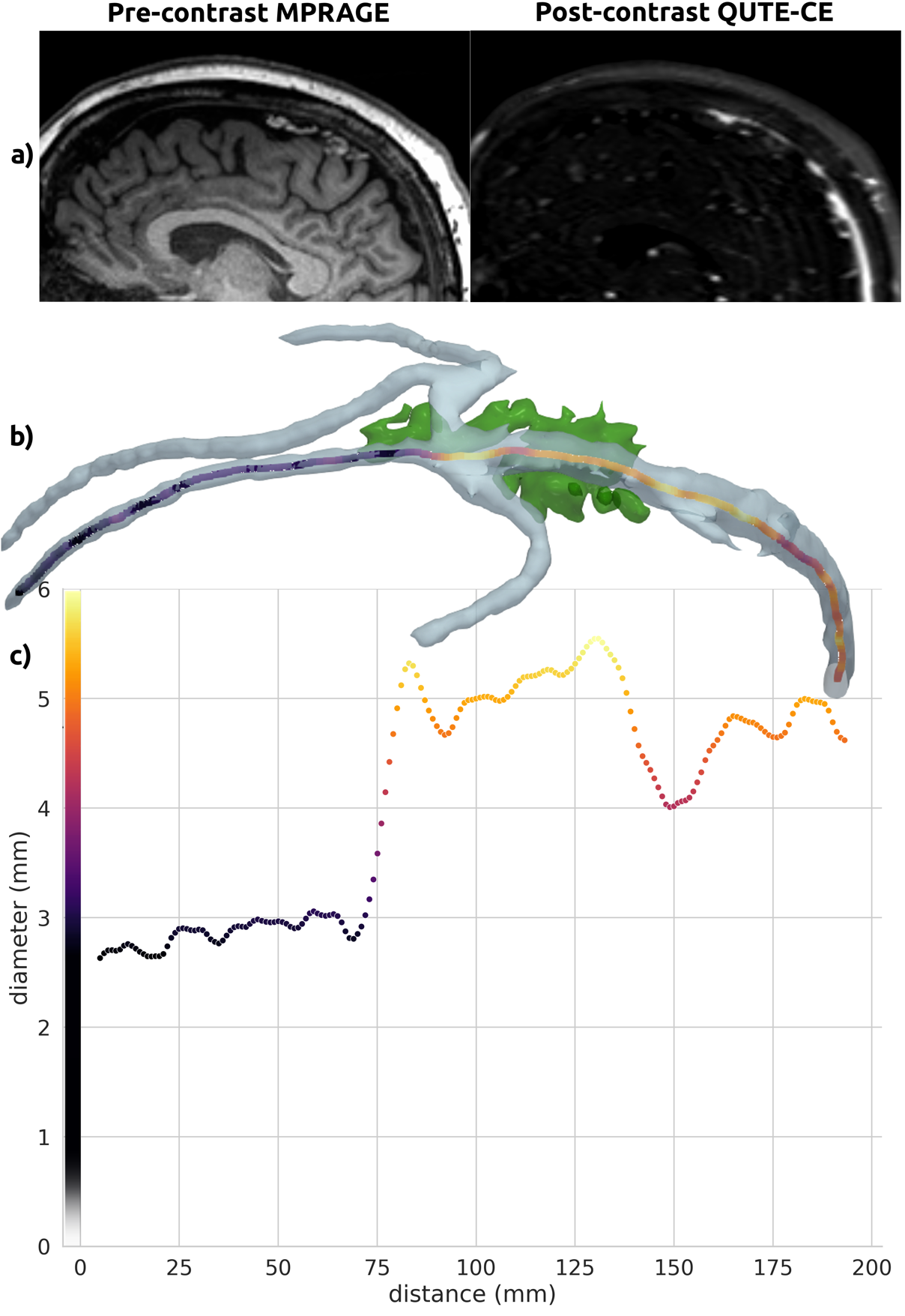
**

**Fig. S4. Diameters of vessel segments of interest can be quantitatively assessed.** **(a)** A pre-contrast Magnetization Prepared RApid Gradient Echo (MPRAGE) image showed intrinsic T1 hyperintensity which likely represents the ossification along the falx in one participant. The post-contrast Quantitative Ultrashort Time-to-Echo Contrast-Enhanced MR angiography (QUTE-CE MRA) captured the lumen of the potentially affected vessel segment. **(b)** Semi-transparent blue area shows the rendering of the segmented superior sagittal sinus from the QUTE-CE MRA and green area shows the possible ossification along the falx from MPRAGE. A centerline is calculated through the potentially affected area, with each point colored according to the measured diameter (color bar in c). **(c)** The plot of the rolling average of the measured diameter at each point from the anterior tip of the superior sagittal sinus to its posterior base. Points are colored according to diameter to enable easy visual comparison between c and b.

**Video S1.**

[**https://youtu.be/zTo27UXCG5U**](https://youtu.be/zTo27UXCG5U)

**3D Rendering.** A rotating 3D rendering of the cerebrovasculature using a QUTE-CE post-contrast image. The brain has been cropped from an image of the whole head to enable closer examination of the neurovascular details captured by the technique. Both arteries and veins are captured with high contrast enabling detailed examination directly from the post-contrast intensity image. This video accompanies the brain rendering in Fig. 1 but displays the full 3D nature of the image.

Video S2.

<https://youtube.com/shorts/CQVOgq3vfWE?feature=share>

**Dynamic Thresholding.** Video demonstrating the range of vascular detail available across the wide dynamic range of QUTE-CE MRA intensity values. Video is produced with image data from a single post-contrast scan and rendered in 3D Slicer. The “color opacity mapping” bar from 3D Slicer is included in the upper right corner. This bar captures the changes to the intensity visualization threshold throughout the video. It begins with a relatively low threshold to render all vascular intensity. Then, the visibility threshold is incrementally increased until only the highest intraluminal blood intensities remain, corresponding to the largest vessels. The application of a simple thresholding approach thus enables examination across the range of vasculature captured at each point in this video. This video accompanies the threshold renderings in Fig. 8 but captures far more intermediate points. Note: the subject has been defaced in this rendering for privacy leading to seemingly disconnected vessels leading into and out of the front of the head. These vessels are fully present in the original data.
